# What can't colostrum do? Exploring the effects of supplementing colostrum after the first day of life: A narrative review*

**DOI:** 10.3168/jdsc.2024-0708

**Published:** 2024-12-20

**Authors:** David L. Renaud, Michael A. Steele

**Affiliations:** 1 Department of Population Medicine, University of Guelph, Guelph, ON, Canada N1G 2W1; 2 Department of Animal Biosciences, University of Guelph, Guelph, ON, Canada N1G 2W1

## Abstract

The objective of this narrative review is to explore the nontraditional uses of colostrum in dairy calves beyond the first and second feeding after birth. Colostrum is well established as a crucial component of early life management in dairy calves, providing essential antibodies for passive immunity. However, recent studies suggest that extending colostrum supplementation beyond the initial feedings, as well as incorporating transition milk, can yield benefits for calf health and development. Research indicates that such supplementation may lead to enhancements in gut development, improved health scores, and reduced incidence of diseases such as diarrhea and respiratory disease. Furthermore, recent findings have highlighted colostrum's potential to aid in the recovery from diarrhea and support calves during the weaning phase. Despite these findings, the exact mechanisms behind these benefits are still unclear, necessitating further research. Additionally, studies with larger sample sizes and varied housing conditions are needed to fully elucidate colostrum's benefits beyond the first day of life.

## Highlights

• Extended colostrum or transition milk supplementation boosts gut health, growth, and immunity.
• Colostrum supplementation may aid in diarrhea recovery and boost growth during weaning.
• More research is needed in varied conditions with iso-energetic, iso-nitrogenous diets.

## Graphical Abstract

**Figure:**
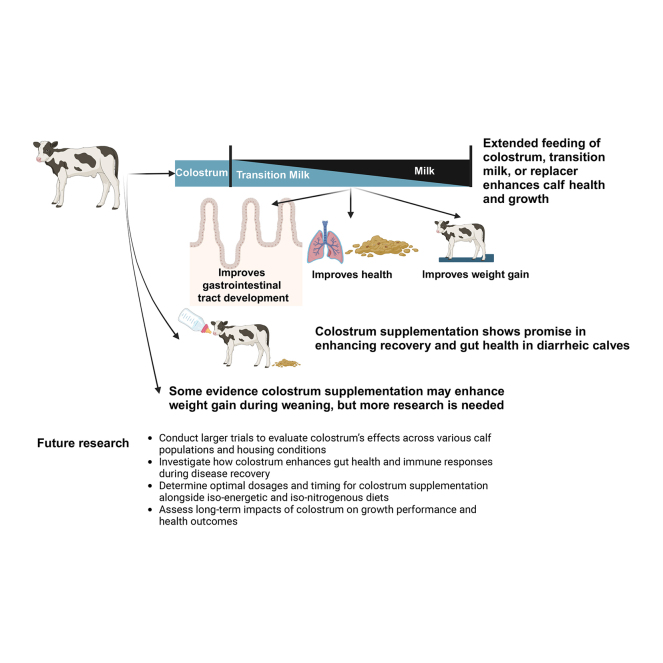
**Summary:** Colostrum is essential for newborn dairy calves, providing antibodies that support early immunity. Beyond the first feedings, extending colostrum or transition milk supplementation can offer additional benefits for calf health and growth. Research shows it may improve gut development, reduce diseases such as diarrhea, and improve weight gain. Colostrum also shows promise in helping calves recover from diarrhea and supporting growth during weaning, although more studies are needed to confirm these effects. Future research should investigate how colostrum works in different housing systems, the best timing and dosage for supplementation, and its long-term impact on calf health and development.

Colostrum is a cornerstone of early calf management. The importance of the first colostrum feeding to the health of calves was well established by 1937 (Kertz et al., 2017); however, the basic principles of feeding colostrum from dams to their offspring to establish successful transfer of passive immunity were identified between the late 1970s to mid 1980s. This includes feeding a sufficient quantity (10% BW) of high-quality (≥50 g/L of IgG; <100,000 cfu/mL of total bacteria) colostrum quickly after birth (Godden et al., 2019). Following these principles, it should be possible to prevent the development of failed transfer of passive immunity, which has been shown to increase the risk of diarrhea, respiratory disease, and mortality (Raboisson et al., 2016).

The importance of colostrum during the first day of life is unquestionable, but there is growing interest in its additional benefits beyond this period, particularly with extended feedings of colostrum or transition milk (defined as milkings 2 to 6 after calving; Godden, 2008) after the initial 24 h of life, during disease episodes, or around weaning challenges. Recent studies suggest that supplementing colostrum or transition milk in these contexts may enhance calf health and performance, supporting calves during high-risk periods when they are especially vulnerable to setbacks. Hence, the objective of this narrative review is to explore nontraditional uses of colostrum in dairy calves beyond the first 24 h of life. Specifically, we will explore colostrum's potential for supplementation during the first weeks of life, its role in disease treatment, and its application during the weaning phase.

After colostrum production, cows naturally continue to produce milk that differs significantly from mature milk (Figure 1). Although neonatal intestinal IgG absorption declines rapidly after 12 h (Matte et al., 1982), IgG and other immunoglobulins can still influence intestinal development and immune function both locally and systemically. While IgG has traditionally been the focus of colostrum research, other bioactive components, such as lactoferrin, insulin-like growth factor, insulin and glucagon-like peptide, and the unique nutritional profile of colostrum and transition milk, which includes higher levels of fat and protein and lower levels of lactose, likely play crucial roles in promoting calf growth, immunity, and gastrointestinal development (Fischer-Tlustos et al., 2021). Specifically, the fat in colostrum and transition milk is vital for the metabolism and thermoregulation of newborn calves (Quigley and Drewry, 1998), and certain fatty acids enhance immune response during the initial weeks of life (Contarini et al., 2014; Opgenorth et al., 2020). Additionally, colostrum and transition milk contain high levels of oligosaccharides, which likely aid in establishing a healthy microbiome (Fischer et al., 2018). Hormones, such as insulin and IGF-I, promote gastrointestinal cell proliferation and support intestinal growth (Fischer-Tlustos et al., 2021). These improvements in gut structure and immune regulation likely contribute to the benefits observed with extended supplementation of colostrum, colostrum replacer, or transition milk beyond the initial feeding discussed below.

**Figure 1:**
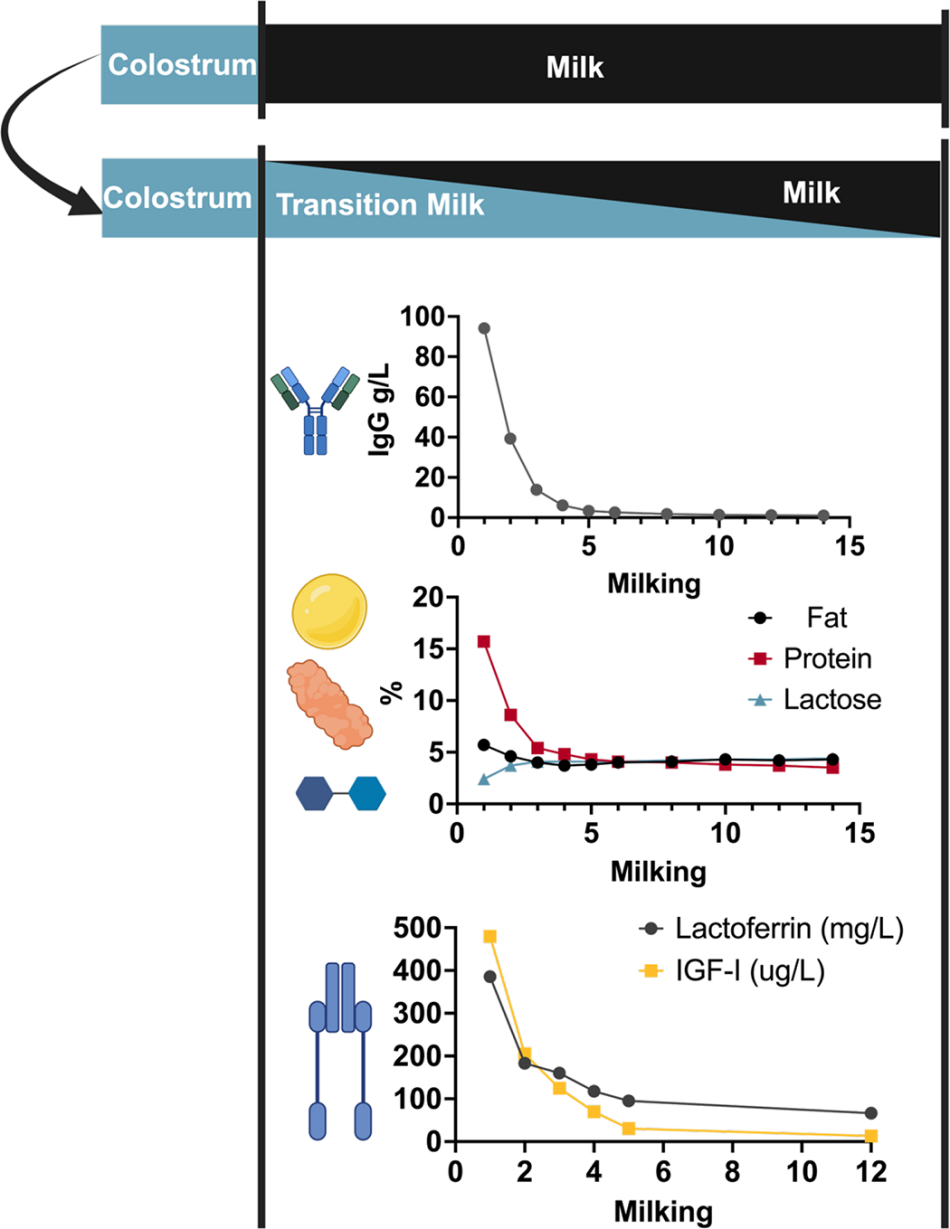
Composition of transition milk with the levels of IgG, fat, protein, lactose, lactoferrin, and IGF-1 shown over time. Data shown from Fischer-Tlustos et al. (2024) and Fischer-Tlustos et al. (2020).

Several studies have explored the benefits of extending colostrum and transition milk feeding beyond the initial postnatal period to support gastrointestinal development and calf health. Van Soest et al. (2022) fed male Holstein calves colostrum at birth, followed by either milk replacer or transition milk (milk from milkings 2 to 4 postcalving) 3 times daily for 4 d. At d 5, calves fed transition milk showed significant intestinal improvements, including 2 times greater villus length and width, a 70% increase in submucosal thickness, and greater mucosal surface area in the ileum and mid jejunum, with no effect on crypt depth. Calves fed transition milk also had 50% more cell proliferation in the crypts and villi, gained 300 g/d more, and exhibited better health scores for cough, feces, nose, and ears compared with those fed milk replacer. Similarly, Pyo et al. (2020) reported that male Holsteins fed colostrum followed by colostrum, whole milk, or a 1:1 colostrum-to-whole-milk mixture (mimicking transition milk) every 12 h from 12 to 72 h post-birth showed increased villus height in the colostrum and mixture groups compared with the whole milk group when euthanized at 75 h of age. The gastrointestinal surface area in the jejunum and ileum was also increased in calves fed colostrum or the mixture compared with the whole milk group. Bühler et al. (1998) also found that offering colostrum for 6 feedings of colostrum over the first 3 d of life led to greater villus circumferences, areas, and heights in the small intestine compared with calves that received a single feeding of colostrum in male calves euthanized at d 8 of life. Finally, Hammon and Blum (1997) demonstrated that calves fed colostrum from the first 6 milkings over the first 3 d of life exhibited greater xylose absorption and higher basal glucose levels compared with those fed only milk replacer, suggesting enhanced absorptive capacity with prolonged colostrum supplementation. Cumulatively, these studies indicate that extended feedings of colostrum or transition milk beyond the initial postnatal period can enhance intestinal morphogenesis and cellular proliferation, although the mechanism for these effects remains unclear.

Beyond these studies evaluating the impact of colostrum or transition milk supplementation on gut development, other research has explored the carryover effects of feeding transition milk or colostrum/colostrum replacer mix within the first few days of life on calf health and growth performance. Conneely et al. (2014) investigated the effects of extended feedings of transition milk after colostrum by randomly assigning calves to receive 0, 2, or 4 feedings of transition milk from the second milking postcalving. Calves that received additional transition milk following the initial colostrum feeding had lower odds of worse eye/ear scores and nasal scores compared with calves receiving whole milk over the 12-wk experimental period. No differences were found between the groups for fecal consistency or cough score, though growth was not assessed in this study. Van Soest et al. (2020) fed calves 2 initial colostrum feedings, followed by 1 of 3 diets ([1] transition milk [second to fourth milking postcalving], [2] a 50:50 blend of milk replacer and colostrum replacer, or [3] milk replacer alone) administered in nine 1.9-L feedings from d 2 to 4 of life. Following this period, all calves were fed and managed similarly. Of the 105 calves enrolled, those fed transition milk or the 50:50 mixture weighed 3 kg more by d 56 compared with those fed only milk replacer. Haptoglobin levels were lower from d 7 to 21 in the transition milk and 50:50 mixture groups, possibly indicating reduced inflammation. Despite these differences, health scores for ears, eyes, and feces were similar across all groups; however, only 17% of calves required treatment and a mortality rate of less than 1% was reported, indicating overall high calf health in the study. Berge et al. (2024) also looked at the impact of supplementing colostrum for 4 d after 2 meals of colostrum. Specifically, calves were assigned to receive 1 L of colostrum or placebo supplement (nutritionally similar to colostrum) in the morning milk replacer feeding 24 h after birth and half a liter of colostrum or placebo in the morning milk replacer feeding for the next 3 d. Body temperatures and respiratory scores were lower in calves supplemented with colostrum compared with control calves in the first 21 d of life. Further, reductions in fecal samples positive for *Cryptosporidium parvum* on d 14 of life were also found in colostrum-supplemented calves; however, no differences were found in growth in the first 4 wk of age. In a study by da Silva et al. (2023), after colostrum feeding, 36 calves were randomly assigned to receive 4 L of milk per day, 4 L/d maternal transition milk, or 4 L/d whole milk with 280 g/d colostrum powder added. All diets were fed for 3 d and then calves were fed 6 L/d whole milk until d 56. Overall, no effects were found on calf health or growth, which may be due to the limited sample size of only 12 calves per treatment group. In summary, several studies have demonstrated the beneficial effects of short-term colostrum and transition milk supplementation on calf health, with improved scores for eye, ear, and nasal conditions, reduced inflammation, and lower incidence of specific pathogens, although growth outcomes varied across studies.

Additional research has explored the effects of long-term supplementation (i.e., >1 wk) of colostrum and transition milk on health and growth outcomes. After a colostrum meal, Kargar et al. (2020) evaluated 3 feeding regimens for Holstein calves: no colostrum with 5 kg of milk, 0.35 kg of colostrum with 4.65 kg of milk daily, or 0.70 kg of colostrum with 4.30 kg of milk daily, over 14 d, followed by 5 kg of milk daily until d 56. Over the 81-d period, calves fed 0.35 and 0.70 kg of colostrum gained 42 and 100 g/d more, respectively, than those fed milk, with colostrum-fed calves also showing greater feed efficiency. Diarrhea incidence was lower in the 0.70 kg group compared with the milk group, whereas both colostrum groups had shorter respiratory disease duration than the milk group. A similar study was completed by Kargar et al. (2021). A total of 84 healthy female Holstein calves were assigned to 1 of 4 treatment groups after 2 colostrum feedings, receiving varying daily amounts of transition milk (0, 0.5, 1, or 2 L) while partially replacing pasteurized waste milk for the first 3 wk of life. Calves fed 2 L of transition milk daily gained more weight than the control group during the postweaning period (60 to 90 d: +0.14 g/d) and overall (0 to 90 d: +0.09 g/d). They also showed improved feed efficiency, lower incidence, and reduced duration of diarrhea compared with the other groups. A study by Snodgrass et al. (1982) housed 42 calves with their dam for 2 d and then allocated them to receive 3 to 4 L of whole milk per day, whole milk with 10% inclusion of pooled colostrum, or whole milk supplemented with 10% of pooled colostrum from cows previously vaccinated against rotavirus. It was found that those fed colostrum from cows that received rotaviral vaccination had a reduced number of days with diarrhea and improved growth compared with calves that received whole milk. Only one study by Nocek et al. (1984) found a negative effect of colostrum feeding. Specifically, they fed colostrum with ≤46 g/L IgG from birth to 96 h, then assigned calves to receive frozen colostrum or 2 different types of milk replacer supplemented with antimicrobials (oxytetracycline and neomycin). All groups received a low plane of nutrition (1.81 L per feeding, twice daily). Overall, BW gain was similar between groups (0.50 kg/d in the colostrum group vs. 0.46 to 0.47 kg/d in the milk replacer groups); however, calves fed colostrum had a higher proportion of days with diarrhea (24% vs. 13% to 16%). No other differences in health were noted.

Several studies have explored the extended supplementation of colostrum replacer beyond the initial days of life. Berge et al. (2009) enrolled 273 Holstein calves (1 to 3 d old) at 3 calf-rearing facilities, assigning them to receive either 70 g of colostrum replacer in milk replacer twice daily for 14 d, 70 g of a placebo supplement (matched levels of fat and protein to the colostrum replacer) in milk replacer twice daily for 14 d, or a control group receiving only milk replacer. Calves fed the colostrum supplement had a lower risk of diarrhea (risk ratio: 0.61; 90% CI: 0.49 to 0.78) and a reduced hazard of antimicrobial treatment (hazard ratio [**HR**]: 0.57; 90% CI: 0.38 to 0.87) in the first 28 d of life compared with controls, with no significant difference found between the placebo and control groups. Growth was also higher in the colostrum group (+0.04 kg/d) in the first 28 d after arrival, and grain intake was greater in both the colostrum and placebo groups relative to the control group. Chamorro et al. (2017) conducted a similar study where 202 1-d-old calves were assigned to receive milk replacer or 150 g of colostrum replacer added to their milk replacer twice daily for the first 14 d after arrival to a calf-rearing facility. The odds of having abnormal fecal, respiratory, depression, and umbilical scores were 0.15, 0.46, 0.21, and 0.18 times, respectively, lower in calves receiving the colostrum compared with control calves. Antimicrobial treatment was also found to be lower (18.8% in colostrum group vs. 76.5% in control group). The average daily gain over the 56-d experimental period was not different between groups. Finally, McCarthy et al. (2024) conducted a study where calves were initially fed colostrum at 0 and 12 h after birth and then assigned to 1 of 4 groups: (1) milk replacer from d 2 to 14, (2) 760 g of colostrum replacer per day on d 2 and 3 along with milk replacer, (3) 90 g of colostrum replacer per day with milk replacer for 14 d, and (4) 760 g of colostrum replacer per day on d 2 and 3 plus 90 g of colostrum replacer mixed with milk replacer from d 4 to 14. Calves receiving colostrum replacer on d 2 and 3, and those fed colostrum replacer for 14 d, had a reduced risk of diarrhea compared with those receiving only milk replacer. Further, during the 49-d experimental period, calves fed colostrum for 14 d had a markedly lower risk of mortality, with a 3.8 times higher hazard of mortality in calves fed solely milk replacer.

Overall, evidence supports the positive effects of extended colostrum feeding, whether for a few days after birth or during the first weeks of life, although outcomes may vary based on feeding protocols and disease burden. However, there are limitations to consider in this literature. Many of the studies mentioned lacked iso-energetic and iso-nitrogenous treatment and control groups, so future research should ensure equal levels of energy and protein to verify whether similar findings emerge. Additionally, while improved intestinal development has been observed, the mechanisms behind many of these results remain unclear. Future studies should investigate changes in immune function, gut function, and microbiota to clarify the reasons for these changes. Last, this body of literature has explored a wide range of feeding regimens, highlighting the need for future research to define the most effective protocol while considering economic constraints.

In humans, bovine colostrum has been used for treatment of diarrhea in children. Specifically, a meta-analysis by Li et al. (2019) showed bovine colostrum supplementation reduced stool frequency in infectious diarrhea by 1.42 times per day, decreased diarrhea occurrence by 71%, and lowered the odds (odds ratio = 0.29) of pathogen detection compared with the placebo group. Further, a meta-analysis of 7 trials by Hałasa et al. (2022) found that bovine colostrum reduced the relative risk of upper respiratory tract infections, highlighting its potential in this context.

Despite evidence from human medicine supporting the therapeutic benefits of colostrum, few trials have evaluated the utility of bovine colostrum for treating diseases in cattle, particularly calves with diarrhea. Carter et al. (2022) evaluated colostrum supplementation at the onset of diarrhea by assigning calves to 1 of 3 treatments: control milk replacer, a 50:50 blend of milk replacer and colostrum replacer for 4 feedings, and the same blend for 8 feedings. Calves receiving the mixture for 8 feedings had a faster resolution of diarrhea (HR: 2.29; 95% CI: 1.31–4.01) and gained 98 g/d more over the 56 d following enrollment compared with controls. In another study, Gamsjäger et al. (2023) challenged calves with *Cryptosporidium parvum* and compared those receiving a 50:50 blend of milk replacer and colostrum replacer once daily, along with 2 additional milk meals, to calves receiving 2 L of milk replacer 3 times daily. Daily supplementation with colostrum mitigated some clinical signs of diarrhea (i.e., fewer signs of depression and fever), reduced the need for oral electrolyte therapy, and positively influenced host gut immune responses. Additionally, challenged calves that received colostrum exhibited a microbiome profile more similar to that of unchallenged calves, suggesting a beneficial modulatory effect on gut health. Additionally, Kaçar et al. (2022) demonstrated that maternal colostrum, with or without sodium bicarbonate, combined with paromomycin improved clinical outcomes and fecal oocyst shedding more effectively than paromomycin alone. Proteomic analysis revealed that colostrum-treated calves experienced positive shifts in key immune-related proteins, with the most pronounced effects in calves supplemented with colostrum and sodium bicarbonate. Collectively, these studies highlight the therapeutic potential of colostrum supplementation for improving recovery and gut health in diarrheic calves.

With respect to respiratory disease, there is a paucity of literature evaluating the utility of colostrum as a therapy. A single randomized clinical trial evaluated the effectiveness of colostrum replacer compared with a placebo milk replacer in reducing disease bouts in dairy calves following a triggered alarm for decreased milk intake or drinking speed (Cantor et al., 2021). A total of 84 calves (42 in each group) were enrolled when they triggered the alarm between d 14 and 50. The calves received either 125 g of colostrum replacer or a placebo for 3 d and were monitored for bovine respiratory disease and diarrhea. Calves that received colostrum had 1.64 times lower odds of developing respiratory disease and 1.50 times lower odds of having lobar lung consolidation in the week following intervention compared with placebo calves. However, there was no significant difference in diarrhea likelihood or ADG between the 2 groups.

These studies demonstrate that colostrum supplementation can accelerate recovery and promote weight gain in calves when used as a supportive therapy for diarrhea and, in a single study, reduce the odds of respiratory disease in calves exhibiting altered drinking behavior. More extensive trials are needed, however, that include larger sample sizes and diverse housing conditions to fully elucidate the therapeutic potential of bovine colostrum in calf health management.

Bovine colostrum has long been used during stressful periods, including weaning, in other species. Specifically, in piglets, supplementation of bovine colostrum replacer for 12 to 14 consecutive days at weaning has been shown to enhance intestinal lymphocyte density, villus height, and overall intestinal integrity, leading to improved feed intake, weight gain, and gain-to-feed ratio (King et al., 2008; Huguet et al., 2012). Only a single study has evaluated the supplementation of colostrum during the period of weaning in calves (Edwards et al., 2024). In this randomized trial, starting at weaning (57 d of life), calves were assigned to receive 3.8 L of milk replacer or a mixture of 3.8 L of milk replacer with 125 g of colostrum replacer once daily. While there were no initial differences in BW, calves receiving colostrum were 2.79 and 2.76 kg heavier than controls by d 77 and 84, respectively, and showed evidence for greater weight gain over the experimental period. However, no significant differences were observed in other health and performance measures. Overall, while colostrum supplementation may improve weight gain during weaning, the underlying mechanism remains unclear, and more studies are needed to replicate this finding.

Colostrum is a crucial component of calf management, with established protocols ensuring effective transfer of passive immunity in the initial hours of life. Recent research has broadened our understanding of colostrum's role, suggesting that supplementation beyond the first feeding may offer additional benefits. Studies indicate that extending colostrum and transition milk feeding can enhance gut development, improve health scores, and reduce disease incidence during early life. Although promising outcomes have been observed, particularly in treating diarrhea and supporting calves during weaning, further research with larger sample sizes and diverse housing conditions is needed to fully elucidate its potential as a therapy. Further, the exact mechanisms contributing to the benefits of continued supplementation of colostrum and transition milk are still unclear, necessitating further research. Overall, continued exploration of nontraditional uses of colostrum could lead to improved calf health and productivity.
